# ^111^In-labeled anti-cadherin17 antibody D2101 has potential as a noninvasive imaging probe for diagnosing gastric cancer and lymph-node metastasis

**DOI:** 10.1007/s12149-019-01408-y

**Published:** 2019-10-12

**Authors:** Kentaro Fujiwara, Atsushi B. Tsuji, Hitomi Sudo, Aya Sugyo, Hiroki Akiba, Hiroko Iwanari, Osamu Kusano-Arai, Kouhei Tsumoto, Toshimitsu Momose, Takao Hamakubo, Tatsuya Higashi

**Affiliations:** 1 Department of Molecular Imaging and Theranostics, National Institute of Radiological Sciences, National Institutes for Quantum and Radiological Science and Technology (QST-NIRS), 4-9-1 Anagawa, Inage, 263-8555 Chiba, Japan; 2 Laboratory of Pharmacokinetic Optimization, Center for Drug Design Research, National Institutes of Biomedical Innovation, Health and Nutrition, Osaka, Japan; 3 Department of Quantitative Biology and Medicine, Research Center for Advanced Science and Technology, The University of Tokyo, Tokyo, Japan; 4 Institute of Immunology Co., Ltd., Tokyo, Japan; 5 Department of Bioengineering, School of Engineering, The University of Tokyo, Tokyo, Japan; 6 Department of Radiology, Faculty of Medicine, International University of Health and Welfare, Chiba, Japan; 7 Department of Protein-Protein Interaction Research, Institute for Advanced Medical Sciences, Nippon Medical School, Tokyo, Japan

**Keywords:** Cadherin-17, SPECT, Radiolabeled antibody, Gastric cancer, Lymph-node metastasis

## Abstract

**Objective:**

Cadherin-17 (CDH17) is a transmembrane protein that mediates cell–cell adhesion and is frequently expressed in adenocarcinomas, including gastric cancer. CDH17 may be an effective diagnostic marker for the staging of gastric cancer. Here, we developed an ^111^In-labeled anti-CDH17 monoclonal antibody (Mab) as an imaging tracer and performed biodistribution and single-photon emission computed tomography (SPECT)/computed tomography (CT) imaging studies using mice with CDH17-positive gastric cancer xenografts. CDH17 expression in gastric cancer specimens was also analyzed.

**Methods:**

The cross-reactivity and affinity of our anti-CDH17 Mab D2101 was evaluated by surface plasmon resonance analysis and cell enzyme-linked immunosorbent assay, respectively. Biodistribution and SPECT/CT studies of ^111^In-labeled D2101 (^111^In-D2101) were performed. CDH17 expression in gastric cancer specimens was evaluated by immunohistochemistry.

**Results:**

Surface plasmon resonance analysis revealed that D2101 specifically recognizes human CDH17, but not murine CDH17. The affinity of D2101 slightly decreased as a result of the radiolabeling procedures. The biodistribution study revealed high uptake of ^111^In-D2101 in tumors (maximum, 39.2 ± 9.5% ID/g at 96 h postinjection), but low uptake in normal organs, including the stomach. Temporal SPECT/CT imaging with ^111^In-D2101 visualized tumors with a high degree of tumor-to-nontumor contrast. Immunohistochemical analysis revealed that, compared with HER2, which is a potential marker of N-stage, CDH17 had a higher frequency of positivity in specimens of primary and metastatic gastric cancer.

**Conclusion:**

Our ^111^In-anti-CDH17 Mab D2101 depicted CDH17-positive gastric cancer xenografts in vivo and has the potential to be an imaging probe for the diagnosis of primary lesions and lymph-node metastasis in gastric cancer.

## Introduction

Gastric cancer is the third leading cause of cancer-related death worldwide [1, 2]. Lymph-node (LN) metastasis staging (N-staging) indicates the degree of cancer spread to regional LN and is an important factor involved in treatment planning, such as decisions regarding neoadjuvant chemotherapy, for the management of gastric cancer. N-staging is based on the measurement of LN size with endoscopic ultrasound (EUS) and computed tomography (CT) [2]. An LN diameter of ≥ 10 mm is the diagnostic criterion for LN involvement [3]. Mönig et al., however, reported that LN size is not a reliable indicator of LN metastasis in gastric cancer patients, because 55% of measured metastatic LNs are ≤ 5 mm in diameter [4, 5].

Single-photon emission computed tomography (SPECT) and positron emission tomography (PET) are noninvasive imaging techniques for tumor diagnosis, and have high sensitivity [6]. Although ^18^F-fluoro-2-deoxy-d-glucose (FDG) PET/CT was expected to improve staging through increased detection of involved LN [7, 8], it is not always informative, because ^18^F-FDG is not tumor-specific and sometimes shows false negatives due to low glucose metabolism or false positives due to inflammation [2]. False negatives and false positives could decrease diagnostic accuracy and misdirect treatment planning. Therefore, a tumor-specific tracer that can detect LN metastasis to decrease both false negatives and false positives and improve treatment planning is highly desirable.

Considering that antibodies have high sensitivity and that radiolabeled antibodies recognize their target antigens on the tumor cell surface and accumulate in tumors in vivo, images using radiolabeled antibodies achieve high tumor-to-nontumor contrast [6]. Therefore, immuno-SPECT and immuno-PET have the potential to be highly sensitive and specific diagnostic tools for LN metastasis.

Cadherin-17 (CDH17) is a membrane protein that mediates cell–cell adhesion and is frequently expressed in adenocarcinomas such as gastric cancer, colorectal cancer, and pancreatic cancer [9, 10]. The positive ratio of CDH17 is approximately 60% in both primary and metastatic gastric cancer, suggesting that CDH17 is a promising marker of gastric cancer [11]. Although CDH17 is expressed in human intestinal and pancreatic ductal epithelial cells, it is not found in healthy stomach or LN [12]. Therefore, CDH17 could be a superior target protein for gastric cancer-specific imaging.

We previously generated monoclonal antibodies (Mab) recognizing the extracellular domain of CDH17 [13]. In vitro assays using a gastric cancer cell line AGS revealed a Mab D2101 binds to the antigen on the membrane of living cells with high affinity [13]. D2101, therefore, has the potential of an agent for CDH17-targeted noninvasive imaging. In the present study, D2101 was radiolabeled with ^111^In (^111^In-D2101), and the pharmacokinetics of ^1111^In-D2101 were evaluated by biodistribution studies in CDH17-positive and CDH17-negative gastric cancer xenograft mice. SPECT/CT imaging using ^111^In-D2101 was then performed to confirm its value as an imaging agent. And then, CDH17 expression in primary and metastatic gastric cancer specimens was evaluated by immunohistochemical analysis to clarify the potential of CDH17 as an N-stage marker.

## Materials and methods

### Cell culture and animal models

AGS cells (CRL-1739) and MKN74 cells (JCRB0255) were obtained from ATCC (Manassas, VA, USA) and the Japanese Collection of Research Bioresources Cell Bank (Osaka, Japan), respectively. In a previous study, we isolated an AGS cell clone with high CDH17 expression levels from the parental AGS cell line [13]. The isolated AGS cells have high CDH17 expression (200,000 molecules per cell) [13]. MKN74 cells do not express CDH17 and were used as a negative control [13]. AGS cells were cultured in RPMI-1640 medium (Wako Pure Chemical Industries, Osaka, Japan) supplemented with 15% fetal bovine serum (Gibco, Tokyo, Japan), 1% penicillin–streptomycin mixed solution (Nacalai Tesque, Tokyo, Japan), 1.25 mM sodium pyruvate (Sigma-Aldrich, Tokyo, Japan), and 60 mg/L gentamicin reagent solution (GIBCO, Tokyo, Japan) at 37 °C in a humidified atmosphere with 5% CO_2_. MKN74 cells were cultured in RPMI-1640 medium (Wako Pure Chemical Industries, Osaka, Japan) supplemented with 10% fetal bovine serum (Gibco, Tokyo, Japan) and 1% penicillin–streptomycin solution (Nacalai Tesque) at 37 °C in a humidified atmosphere with 5% CO_2_. Male BALB/c nude mice (5 weeks of age) were purchased from Clea Japan, Inc. (Tokyo, Japan). To generate the tumor-bearing animal models, AGS cells (3.0 × 10^6^ cells in 300 µL/mouse) were inoculated subcutaneously into the right flanks of nude mice. The mice were used 8–9 weeks after inoculation in the following in vivo experiments. MKN74 cells (2.0 × 10^6^ cells/mouse) were inoculated subcutaneously into the right flanks of nude mice. The mice were used 3 weeks after inoculation in the following in vivo experiments. All animal studies were approved by the Animal Care and Use Committee of the National Institute of Radiological Sciences (07-1064-24), and all animal experiments were conducted in accordance with the guidelines regarding animal care and handling.

### Antibodies

Monoclonal antibodies against human CDH17 (D2101 and D2111) were generated as previously described [13]. D2101 was radiolabeled with ^111^In and used for biodistribution and SPECT/CT studies. D2111 was employed for immunohistochemistry, because D2101 is unfitted for the staining technique and D2111 recognizes the same epitope. The subclass of these antibodies was identified as IgG_1_ using the Mouse Typer Sub-Isotyping kit (Bio-Rad, Hercules, CA). The cross-reactivity of D2101 and D2111 was not investigated in our previous study [13]. Therefore, in the present study, we evaluated the cross-reactivity of human and murine CDH17 by surface plasmon resonance analysis.

### Surface plasmon resonance analysis

**Fig. 1:**
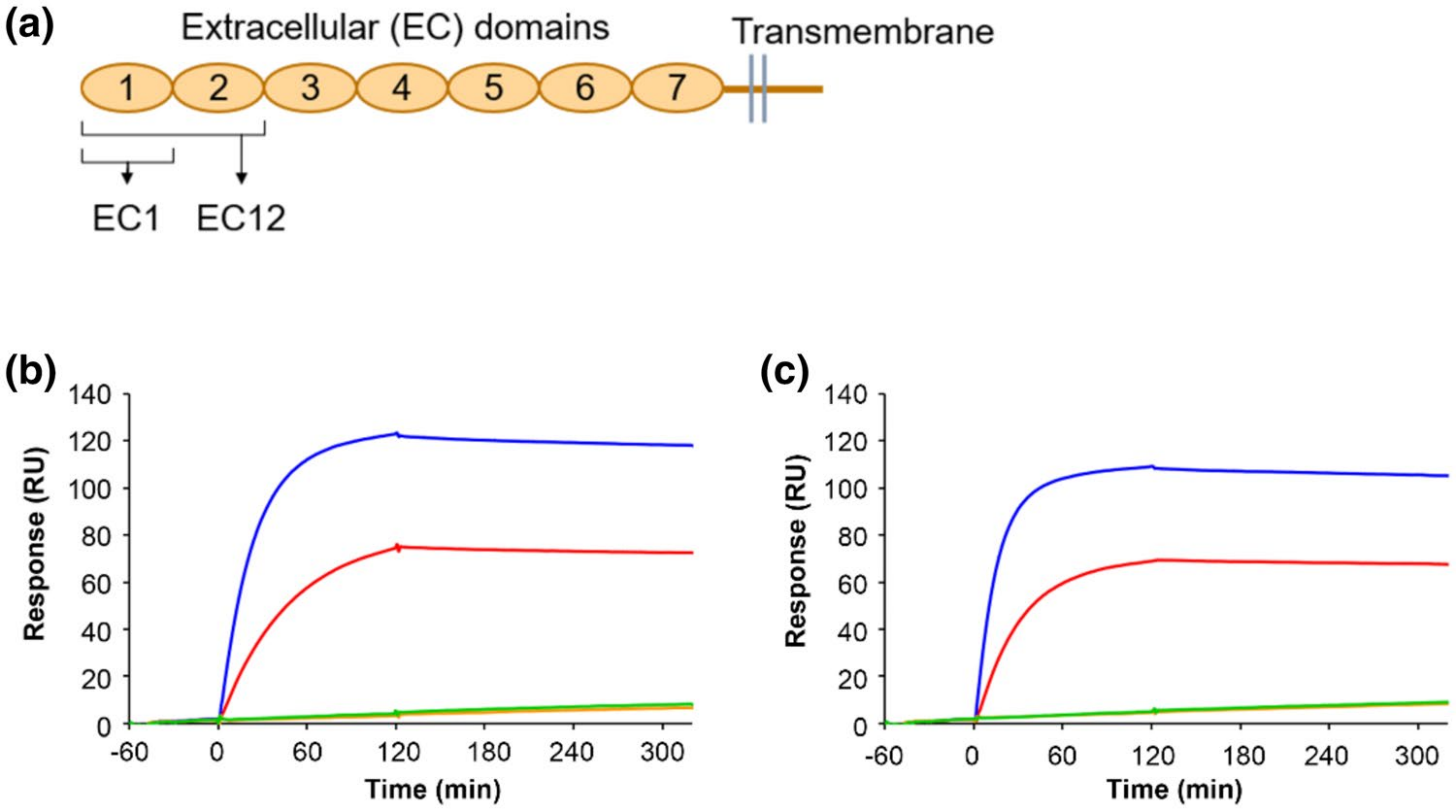
Cross-reactivity of anti-CDH17 antibodies. **a** Design of the CDH17 fragment polypeptides (EC1 and EC12) for the binding analysis. Interaction of D2101 (**b**) and D2111 (**c**) with EC1 and EC12 from human and mouse CDH17 analyzed by surface plasmon resonance. Human EC1: red line, human EC12: blue line, mouse EC1: orange line, and mouse EC12: green line

The cross-reactivity of D2101 and D2111 was evaluated by surface plasmon resonance analysis using recombinant proteins with a partial sequence of CDH17. Genes encoding human and mouse CDH17 were synthesized by GenScript (Tokyo, Japan). Genes of the first ectodomain (EC1) or the first and second ectodomains (EC12) were subcloned into pET-28b between the NcoI and XhoI restriction sites to produce polypeptides bearing the C-terminal hexahistidine tag (Fig. 1a). Genes for human EC1 (huEC1) and human EC12 (huEC12) were determined to encode amino acids 23–128 and 23–244 in UniProt ID Q12864; genes for mouse EC1 (muEC1) and mouse EC12 (muEC12) were determined to encode amino acids 22–127 and 22–243 in UniProt ID Q9R100. The expression and purification protocols were the same as those published previously [14] with a modification of the purification after separation of the proteins by immobilized metal-ion affinity chromatography; the eluate was directly purified by size-exclusion chromatography in a HiLoad 26/600 Superdex 75 column (GE Healthcare, Little Chalfont, UK) for EC1 or a HiLoad 26/600 Superdex 200 column (GE Healthcare) for EC12 using a buffer containing 10 mM HEPES, 150 mM NaCl, and 3 mM CaCl_2_ with a pH of 7.5.

Surface plasmon resonance was detected using a Biacore T200 (GE Healthcare) in buffer containing 10 mM HEPES, 150 mM NaCl, 3 mM CaCl_2_, and 0.05% Tween 20, with a pH of 7.5. Both Mabs (D2101 and D2111) were captured on a CM5 sensor chip using a mouse antibody capture kit (GE Healthcare) according to the manufacturer’s protocol. After immobilization of the anti-mouse IgG, 5 μg/mL D2101 or D2111 were run for 120 s, and the buffer was run for 200 s to remove non-specific binders. The association and dissociation of CDH17 fragments (100 nM in the running buffer) were monitored for 120 s and 200 s, respectively.

### Chelate conjugation and radiolabeling

In total, 2.5 equivalents of [(*R*)-2-amino-3-(4-isothiocyanatophenyl)propyl]-*trans*-(*S*,*S*)-cyclohexane-1,2-diamine-pentaacetic acid (CHX-A″-DTPA, Macrocyclics, Dallas, TX, USA) in 50 mM borate buffer (pH 8.5) were added to a solution of D2101 in 50 mM borate buffer. The mixture was incubated overnight at 37 °C, and non-conjugated chelators were removed on a Sephadex G-50 column (GE Healthcare) via elution with 0.1 M sodium acetate buffer (pH 6.0; 730 × *g* for 2 min). CHX-A″-DTPA-conjugated D2101 (DTPA-D2101) in 0.1 M sodium acetate buffer was incubated with a mixture of ^111^InCl_3_ (Nihon Medi-Physics, Tokyo, Japan) and 1 M sodium acetate buffer for 30 min at room temperature. The radiolabeled antibody was purified on a Sephadex G-50 column, and the radiochemical yield was approximately 80%. The radiochemical purity was determined by radio-TLC, and the conjugation ratio of DTPA and the antibody was estimated from the ratio of the ^111^In-DTPA-antibody and ^111^In-DTPA via isoelectric focusing. Size-exclusion high-performance liquid chromatography (HPLC) was performed on an HPLC system (Gilson, Middleton, WI) with an RI detector (Gabi, Raytest, Straubenhardt, Germany) equipped with a Zenix SEC-300 (3 μm, 300 Å, 7.8 × 150 mm; Sepax Technologies, Newark, DE, USA). Phosphate buffer (0.1 M, pH 6.8) flowed through the system at a rate of 1.0 mL/min.

### Cell enzyme-linked immunosorbent assay (ELISA)

The immunoreactivity of intact D2101 and DTPA-D2101 was evaluated by cell ELISA assay using AGS cells as previously described [13]. The absorbance was measured at 450 nm using a microplate reader (SpectraMax M5; Molecular Devices, San Jose, CA, USA).

### Biodistribution study

AGS xenografted mice were randomly divided into 4 groups (*n* = 3/group). Each mouse was injected with 3.7 kBq of ^111^In-D2101 (10 µg) and killed at 6, 24, 48, or 96 h after injection. The tumor weights of AGS and MKN74 xenografts were 648.8 ± 397 mg and 177.0 ± 50 mg, respectively. CDH17-negative MKN74 xenograft mice were randomly divided into 2 groups (*n* = 3/group). Each mouse was injected with 3.7 kBq of ^111^In-D2101 (10 µg) and euthanized at 24 or 96 h after the injection. The blood, lungs, livers, kidneys, spleens, pancreases, stomachs, intestines, muscles, bones, and tumors were collected and weighed, and radioactivity was measured using a gamma-counter (2470 WIZARD^2^, PerkinElmer, Yokohama, Japan). The percentage of the injected dose per gram of tissue (% ID/g) was calculated for each organ. The data are presented as the mean ± SD.

### SPECT/CT imaging

For SPECT/CT imaging, ^111^In-D2101 (1.85 MBq) was injected via tail vein into mice bearing an AGS tumor or an MKN74 tumor (*n* = 1 per each model). The injected protein dose was approximately 30 µg/mouse. The tumor volumes of AGS and MKN74 xenografts were 368.0 mm^3^ and 180.3 mm^3^, respectively. At 24, 48, and 96 h after the injection, an emission scan was conducted for 15–30 min using a VECTor/CT system with a clustered multi-pinhole high-energy collimator (MILabs, Utrecht, The Netherlands). The SPECT images were reconstructed using a pixel-based ordered-subset expectation maximization algorithm with 2 subsets and 8 iterations on a 0.8-mm voxel grid without attenuation correction. After the SPECT scan, CT scans were acquired with an X-ray source set at 60 kV and 615 µA, and the images were reconstructed using a filtered back-projection algorithm for the cone beam. Using PMOD data analysis software (PMOD Technologies, Zurich, Switzerland), regions of interest (ROIs) were manually drawn over the tumors, and the % ID/g was calculated.

### Immunohistochemistry

CDH17 and HER2 expression frequencies were evaluated with the human gastric cancer tissue microarray ST810c from US Biomax, Inc (Rockville, MD, USA). ST810c has primary gastric adenocarcinoma with matched lymph-node metastatic carcinoma as follows: primary gastric adenocarcinoma, 11 cases of grade 2 and 23 cases of grade 3; lymph-node metastasis, 7 cases of grade 2 and 27 cases of grade 3. The grade of tumors was divided according to the manufacturer’s classification. For CDH17 immunohistochemistry, tissue sections were deparaffinized and rehydrated. Antigen retrieval was performed in 10 mM citric buffer (pH 6.0) for 20 min in a pressure cooker (ALPHAX.KOIZUMI CORP, Souka, Japan). Endogenous peroxidase was quenched with 3% hydrogen peroxide, and the specimens were blocked with Block Ace (DS Pharma Biomedical, Osaka, Japan) for 30 min. The sections were incubated with D2111 (5 µg/mL) overnight at 4 °C and then treated with a secondary antibody (Simple stain MAX–PO; Nichirei, Tokyo, Japan) at room temperature for 30 min. The reaction was visualized by diaminobenzidine (Histofine DAB substrate; Nichirei, Tokyo, Japan). Nuclear staining was performed with hematoxylin. For HER2 immunohistochemistry, the Hercep test (DAKO, Glostrup, Denmark) was used according to the manufacturer’s protocol. The slides were scanned using a NanoZoomer (Hamamatsu Photonics, Hamamatsu, Japan). The positive ratios of CDH17 and HER2 were evaluated in adenocarcinoma sections (primary: grade 2, *n* = 11; grade 3, *n* = 23; and LN metastasis: grade 2, *n* = 7; grade 3, *n* = 27). In this study, moderate-to-strong membranous staining for CDH17 and HER2 was defined as positive.

### Statistical analysis

Data were expressed as the mean ± standard deviation. Means were compared using the Student’s *t* test. *p* values of < 0.05 were considered statistically significant.

## Results

### Surface plasmon resonance analysis

We previously reported that D2101 and D2111 bind to the polypeptide containing the first and second ectodomains (EC12) of human CDH17 (Fig. 1a) [13]. Surface plasmon resonance analysis using the ectodomains as an analyte confirmed that both D2101 and D2111 bind huEC12 with slow dissociation steps (blue lines in Fig. 1b, c). Interaction with the first ectodomain (EC1) of human CDH17 (huEC1) was also observed with a size-dependent reduction in response, which indicated similar binding activity to huEC12 (red lines in Fig. 1b, c). No interaction of the two Mabs with EC1 and EC12 of mouse CDH17 was observed (orange and green lines in Fig. 1b, c, respectively). Therefore, both D2101 and D2111 were determined to have specificity for human EC1 of CDH17 and no cross-reactivity with murine CDH17.

### Radiolabeling and cell ELISA

**Fig. 2:**
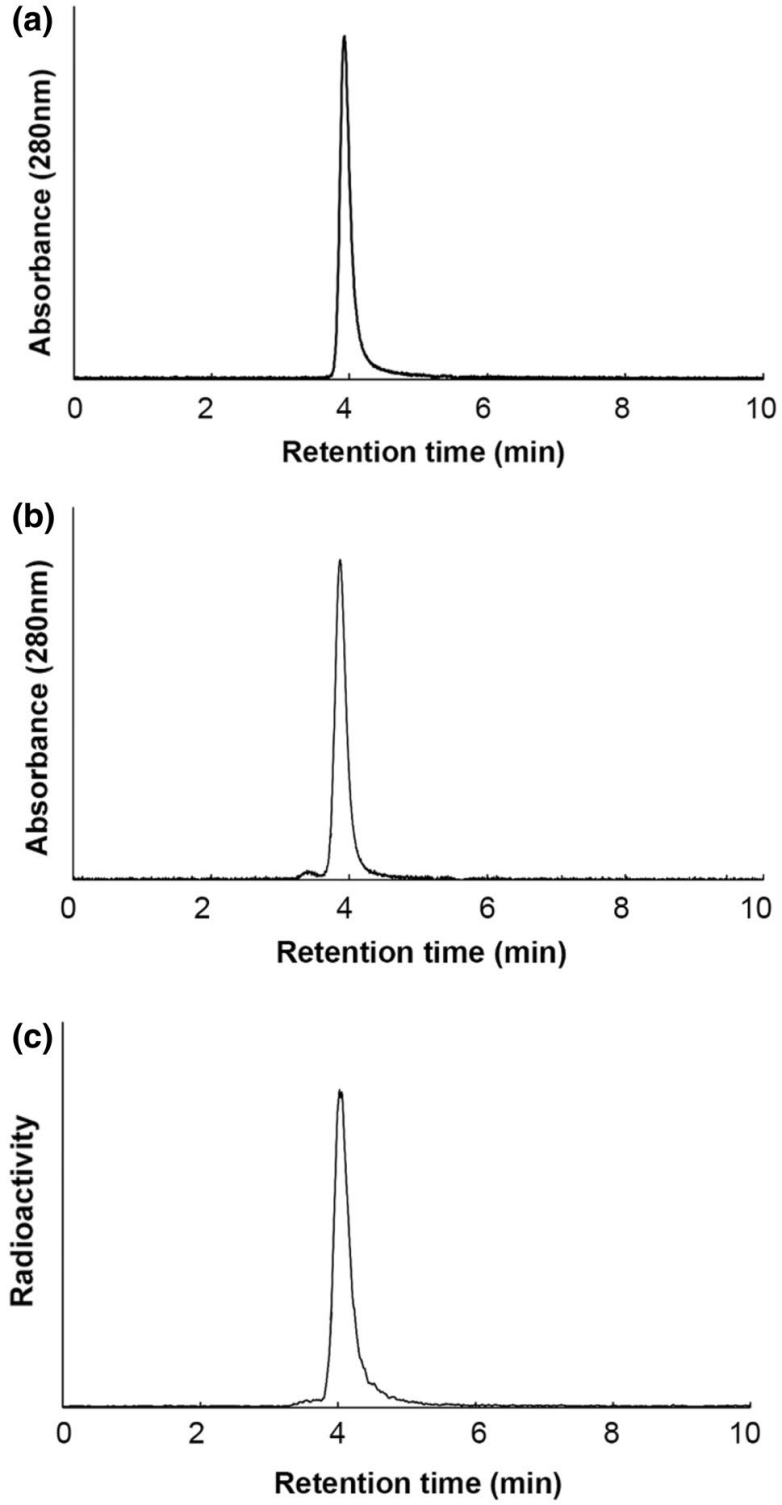
SE-HPLC of anti-CDH17 antibody D2101. Chromatograms of the intact anti-CDH17 antibody D2101 (**a**), DTPA-D2101 (CHX-A″-DTPA-conjugated D2101) (**b**), and ^111^In-D2101 (**c**)

**Fig. 3:**
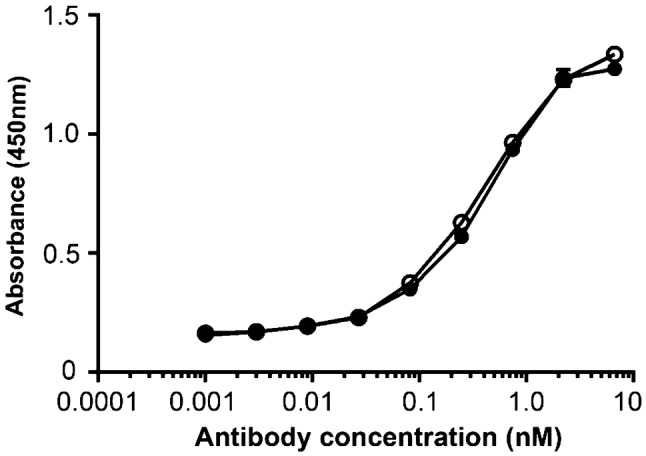
ELISA of intact D2101 and DTPA-D2101 using gastric cancer AGS cells. Intact anti-CDH17 antibody D2101: open circles, DTPA-D2101: filled circles. (*n* = 2 per each point)

The conjugation ratio of chelate to antibody was approximately 0.8, and the radiochemical purity of ^111^In-D2101 was greater than 95%. In the HPLC analysis, the chromatogram peaks of intact D2101, DTPA-D2101, and ^111^In-D2101 were observed at 3.9, 3.9, and 4.0 min, respectively (Fig. 2). The specific activity of ^111^In-D2101 was 51.8 kBq/µg. In the cell ELISA, dose-dependent reactivity of intact D2101 and DTPA-D2101 was observed (Fig. 3). The EC_50_ values of intact D2101 and DTPA-D2101 were 0.289 and 0.326 nM, respectively.

### Biodistribution of ^111^In-labeled D2101

The biodistribution study of ^111^In-D2101 was performed in AGS xenograft mice (Table 1). Tumor uptake of ^111^In-D2101 was 13.6 ± 5.0, 29.8 ± 5.1, 36.1 ± 6.3, and 39.2 ± 9.5% ID/g at 6, 24, 48, and 96 h postinjection (p.i.), respectively. The radioactivity in the blood and normal organs decreased over time (Table 1). The blood activity was 24.0 ± 3.6, 14.7 ± 4.3, 8.0 ± 2.7, and 7.3 ± 3.8% ID/g at 6, 24, 48, and 96 h p.i., respectively. The tumor-to-blood ratios were 0.6 ± 0.3, 2.2 ± 0.8, 4.8 ± 1.2, and 6.4 ± 3.4 at 6, 24, 48, and 96 h p.i., respectively. The uptake of MKN74 xenografts was 5.0 ± 0.1 and 4.8 ± 0.5% ID/g at 24 and 96 h p.i., respectively (Table 2). The tumor uptake significantly differed between AGS xenografts and MKN74 xenografts (*p* < 0.05). Uptake in normal organs of MKN74 xenograft mice was similar or higher than that of AGS xenograft mice.

**Table 1:** Results of the biodistribution study using ^111^In-D2101 in CDH17-positive AGS xenograft mice

Tissue	6 h	24 h	48 h	96 h
Blood	24.0 ± 6.5	14.6 ± 4.3	8.0 ± 2.7	7.3 ± 3.8
Lung	10.1 ± 2.7	5.6 ± 1.5	3.7 ± 1.2	3.7 ± 1.6
Liver	6.2 ± 1.7	4.5 ± 0.7	3.6 ± 1.0	3.6 ± 0.5
Spleen	3.1 ± 0.8	2.7 ± 0.8	1.9 ± 0.4	2.3 ± 1.2
Pancreas	1.8 ± 0.5	1.5 ± 0.6	0.8 ± 0.1	0.7 ± 0.3
Stomach	2.2 ± 0.6	1.6 ± 0.7	0.8 ± 0.2	0.7 ± 0.4
Intestine	2.1 ± 0.6	1.2 ± 0.3	0.7 ± 0.3	0.7 ± 0.3
Kidney	6.8 ± 1.8	4.4 ± 1.0	2.8 ± 0.5	3.5 ± 1.6
Muscle	0.7 ± 0.2	0.9 ± 0.2	0.7 ± 0.3	0.5 ± 0.2
Bone	1.2 ± 0.3	1.6 ± 0.4	1.0 ± 0.4	1.0 ± 0.4
Tumor	13.6 ± 3.7	29.8 ± 5.1	36.1 ± 6.3	39.2 ± 9.5

Values are presented as mean ± SD (%ID/g) (*n* = 3)

**Table 2:** Results of the biodistribution study using ^111^In-D2101 in CDH17-negative MKN74 xenograft mice

Tissue	24 h	96 h
Blood	18.1 ± 0.8	14.8 ± 1.3
Lung	7.1 ± 0.2	5.6 ± 0.4
Liver	7.3 ± 0.1	4.9 ± 0.5
Spleen	5.0 ± 0.5	4.2 ± 1.2
Pancreas	2.0 ± 0.2	1.7 ± 0.7
Stomach	1.7 ± 0.3	1.4 ± 0.1
Intestine	1.7 ± 0.1	1.4 ± 0.2
Kidney	5.1 ± 0.3	4.0 ± 0.6
Muscle	1.1 ± 0.2	1.1 ± 0.1
Bone	1.7 ± 0.1	1.5 ± 0.3
Tumor	5.0 ± 0.1	4.8 ± 0.5

Values are presented as mean ± SD (%ID/g) (*n* = 3)

### SPECT/CT imaging with ^111^In-labeled D2101

**Fig. 4:**
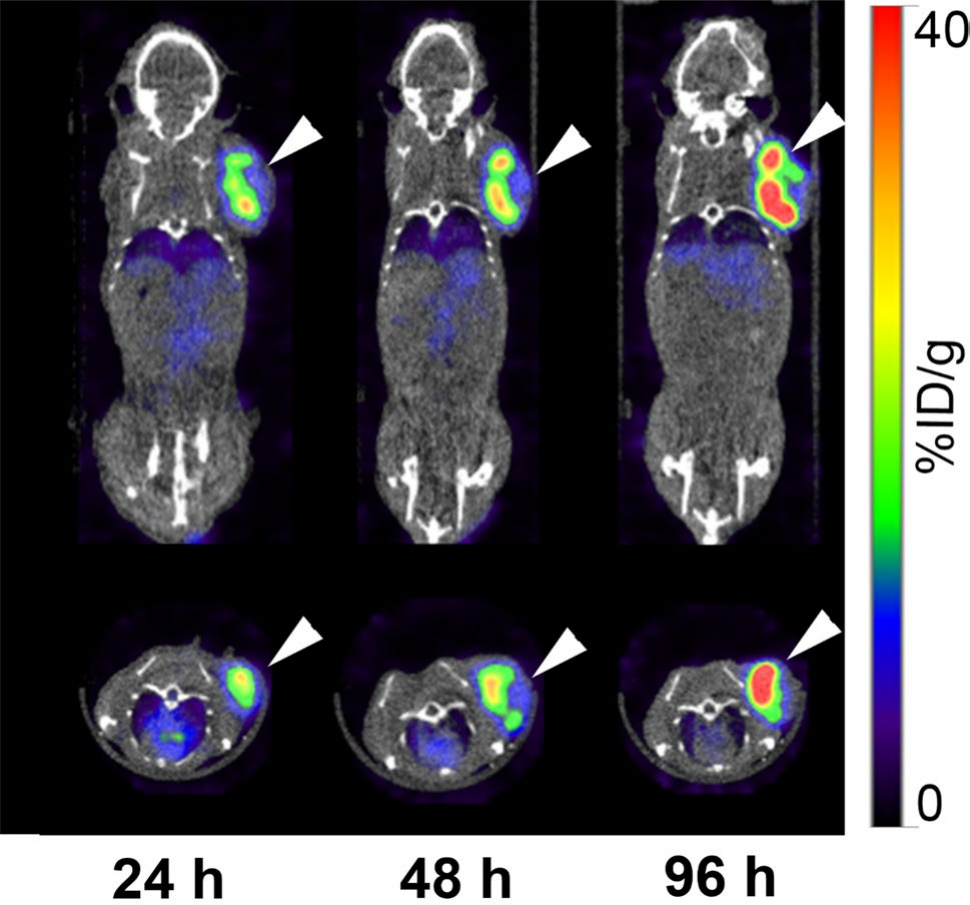
Serial SPECT/CT images of AGS xenograft mice using the ^111^In-labeled anti-CDH17 antibody D2101. Upper panels: coronal images; lower panels: transaxial images. Arrowheads indicate tumors

**Fig. 5:**
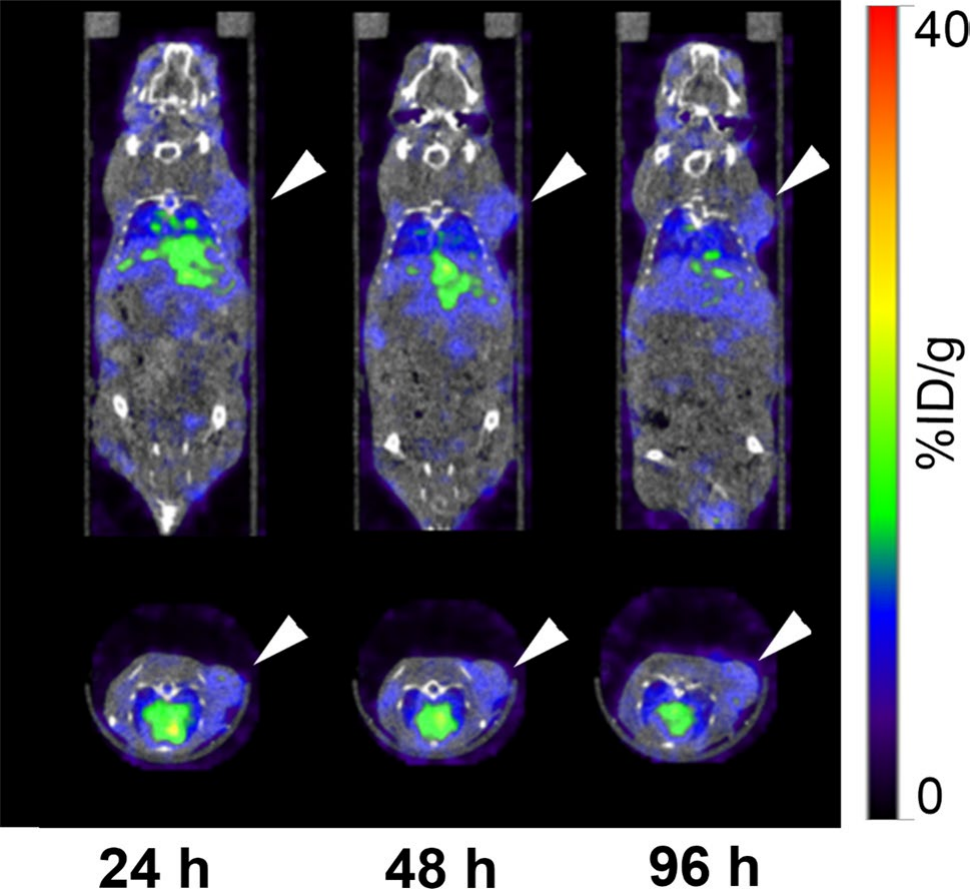
Serial SPECT/CT images of MKN74 xenograft mice using the ^111^In-labeled anti-CDH17 antibody D2101. Upper panels: coronal images; lower panels: transaxial images. Arrowheads indicate tumors

SPECT/CT images of tumor-bearing mice using ^111^In-D2101 are shown in Figs. 4 and 5. The AGS tumor uptake of ^111^In-D2101 increased with time, and the highest tumor accumulation was observed at 96 h p.i. (Fig. 4). Due to the high tumor uptake and low background level, the AGS tumor was visible at 24 h p.i. (Fig. 4). At 24, 48, and 96 h p.i., the AGS tumor uptake was 27.7%, 35.7%, and 39.5% ID/g, respectively. The MKN74 tumor uptake was 7.7%, 7.8%, and 7.7% ID/g at 24, 48, and 96 h p.i., respectively. The uptake patterns were consistent with the biodistribution results reported above.

### CDH17 and HER2 expression analysis in clinical specimens

**Fig. 6:**
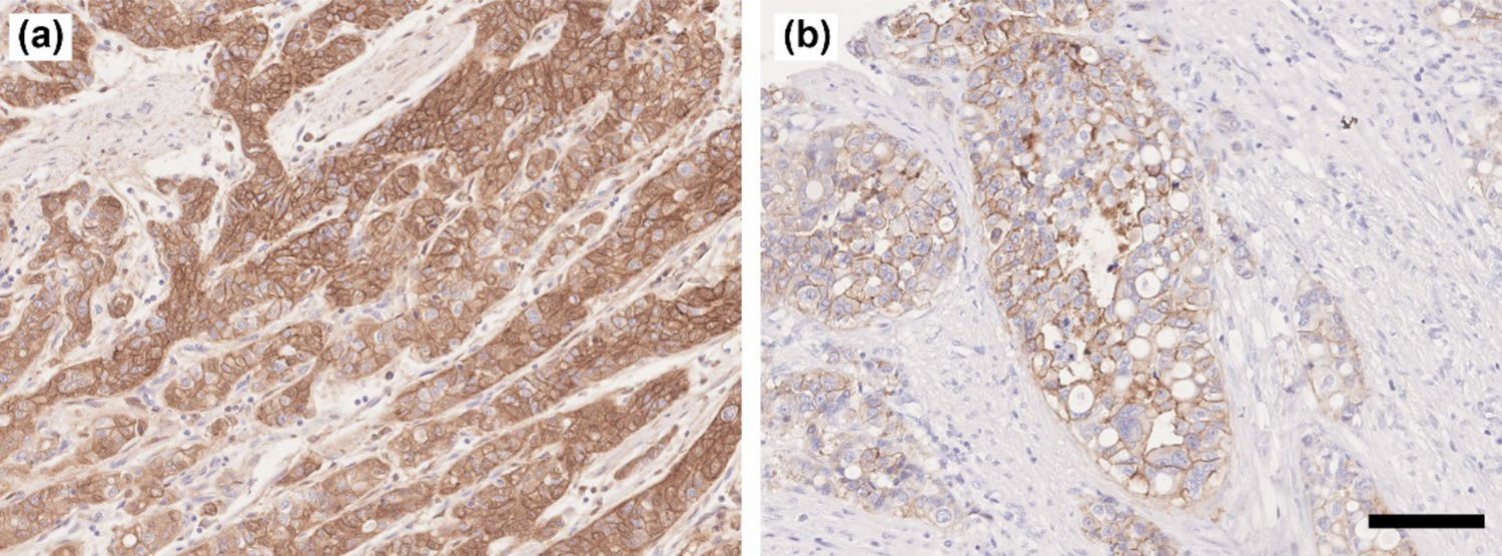
CDH17 and HER2 expression in gastric cancer specimens. **a** Immunohistochemical staining of CDH17 in primary cancer; **b** immunohistochemical staining of HER2 in primary cancer; original magnification: × 200, scale bar 100 µm

The CDH17 and HER2 expression frequencies were evaluated in gastric cancer specimens by immunohistochemistry. CDH17 had strong staining intensity and HER2 had moderate staining intensity (Fig. 6). The positive CDH17 ratios were 63.6% (7/11) for grade 2 and 56.5% (13/23) for grade 3 in the primary cancer specimens, and 86.7% (6/7) for grade 2 and 51.9% (14/27) for grade 3 in the LN metastasis specimens (Table 3). The positive HER2 ratios were 27.3% (3/11) for grade 2 and 8.7% (2/23) for grade 3 in the primary cancer specimens, and 28.6% (2/7) for grade 2 and 7.4% (2/27) for grade 3 in the LN metastasis specimens (Table 3).

**Table 3:** Positive ratio of CDH17 and HER2 in gastric cancer specimens

Disease	CDH17	HER2
Primary		
Grade 2	63.6% (7/11)	27.3% (3/11)
Grade 3	56.5% (13/23)	8.70% (2/23)
LN		
Grade 2	85.7% (6/7)	28.6% (2/7)
Grade 3	51.9% (14/27)	7.41% (2/27)

*Primary* primary gastric cancer, *LN* lymph node metastasis

Table 4 shows the CDH17 and HER2 expression patterns in primary and metastatic cancer specimens. In the CDH17-stained sections, the percentage of CDH17-positive primary cancer and positive LN metastasis [primary (+)/LN (+)] specimens was 80.0% (16/20). The percentage of CDH17-positive primary cancer and CDH17-negative LN metastasis [primary (+)/LN (−)] specimens was 20% (4/20). The percentage of CDH17-negative primary cancer and CDH17-positive LN metastasis specimens was 28.6% (4/14), and the percentage of CDH17-negative primary cancer and CDH17-negative LN metastasis specimens was 71.4% (10/14; Table 4). In the HER2-stained sections, the percentages of primary (+)/LN (−), primary (+)/LN (−), primary (−)/LN (+), and primary (−)/LN (−) cases were 80.0% (4/5), 20% (1/5), 0% (0/29), and 100% (29/29), respectively (Table 4).

**Table 4:** Summary of the CDH17 and HER2 expression patterns in primary and metastatic gastric cancer specimens

Disease	CDH17	HER2
Primary (+)/LN (+)	80.0% (16/20)	80.0% (4/5)
Primary (+)/LN (−)	20.0% (4/20)	20.0% (1/5)
Primary (−)/LN (+)	28.6% (4/14)	0% (0/29)
Primary (−)/LN (−)	71.4% (10/14)	100% (29/29)

*Primary* primary gastric cancer, *LN* lymph node metastasis, + positive, − negative

## Discussion

The present study demonstrated that our anti-CDH17 Mab D2101 radiolabeled with ^111^In has the potential to be an imaging probe for gastric cancer using the CDH17-expressing tumor model AGS cells. The cell ELISA assays showed that the radiolabeling procedures only slightly decreased the binding affinity of D2101, suggesting that ^111^In-D2101 could be used for in vivo imaging. In the biodistribution study, the tumor uptake and tumor-to-blood ratios of ^111^In-D2101 increased over time, reaching their highest values of 39.2% ID/g and 6.4, respectively, at 96 h p.i. The uptake of CDH17-positive AGS xenografts was significantly higher than that of CDH17-negative MKN74 xenografts. This finding suggests that ^111^In-D2101 has specificity for CDH17-positive tumors. The long blood retention time of ^111^In-D2101 is likely due to the functioning of Fc receptors in vascular endothelial cells [15, 16]. The SPECT/CT imaging study confirmed the favorable in vivo kinetics of ^111^In-D2101 and visualized AGS tumors starting at 24 h p.i., although the greatest tumor uptake and tumor-to-blood ratio of ^111^In-D2101 during our period of observation was at 96 h p.i. In contrast, uptake of MKN74 tumors was 7.7% ID/g at 24 h and remained at that level throughout the time-course. This finding is similar to the biodistribution results and demonstrates high and specific uptake of ^111^In-D2101 in CDH17-positive tumors. AGS tumors were visible, and the high tumor uptake of 29.8% ID/g was already observable at 24 h p.i. A shorter waiting time for clear tumor uptake reduces the burden on the patient and enhances the efficiency of the detection method in clinical practice. Therefore, ^111^In-D2101 is a promising imaging agent for detecting CDH17-positive gastric cancer and is applicable to the clinical setting.

In the CDH17 immunohistochemical analysis of gastric cancer specimens, the percentage of CDH17-positive primary cancer and positive LN metastasis [primary (+)/LN (+)] was 80%, suggesting that most metastatic regions are positive for CDH17 when the primary regions are CDH17-positive. This result suggests that CDH17-targeted imaging is expected to be highly accurate for determining the N-stage in gastric cancer. Table 3 shows the antigen-positive ratio in each grade (tumor differentiation). Our results showed that the CDH17 and HER2-positive ratios of grade 3 specimens (poorly differentiated) seemed to be lower than those of grade 2 specimens (moderately differentiated). Especially in HER2, the positive ratio of grade 3 specimens was markedly lower than that of grade 2 (primary: 27.3% for grade 2 and 8.7% for grade 3; LN: 28.6% for grade 2; and 7.4% for grade 3), and the similar findings are already reported: the intestinal type (well-differentiated) gastric cancer exhibited high HER2-positive ratio compared with diffuse (poorly differentiated) type gastric cancer [17–19]. Our results are in line with these reports. In contrast, the difference of CDH17-positive ratios between grades 2 and 3 was not large (primary: 63.6% for grade 2 and 56.5% for grade 3, LN: 85.7% for grade 2 and 51.9% for grade 3) compared with that of HER2. The sample size in the present study is limited and there is no report of large-scale CDH17 expression analysis in cancer specimens. It is required further validation of the correlation between histopathological grade and CDH17-positive ratio with big size samples. In grade 3 cancer, the CDH17-positive ratio was higher than the HER2-positive one. Therefore, CDH17-targeted imaging could more detect cancer compared with HER2-targeted imaging.

The reason for the difference between primary (+)/LN (−) specimens and primary (−)/LN (+) specimens is unclear, although there are several potential explanations: (1) CDH17 expression in cancer cells may change after metastasis, (2) some specimens may not contain CDH17-positive regions due to tumor heterogeneity, and (3) metastasized tumor cells may be CDH17-negative due to the tumor heterogeneity and branched evolution [20]. Further studies are needed to clarify the validity of these possibilities.

The N-stage of gastric cancer critically affects the planning of therapeutic strategies, such as the choice of surgery with neoadjuvant chemotherapy, which is recommended for patients with T3 and T4 stages, and node-positive tumors [2]. Sentinel LN biopsy and EUS-guided fine-needle aspiration are highly accurate methods of determining the N-stage, but they are invasive [21, 22]. Hence, EUS and CT are generally performed as noninvasive methods depending on the size of the LNs [2]. The sensitivity, specificity, positive predictive value, and NPV of CT are 70.8%, 61.9%, 68%, and 65%, respectively [8]. An NPV of 65% indicates that 35% of node-positive patients are “node-negative”, i.e., 35% patients do not receive the optimal treatment. Improving the NPV is, therefore, important for selecting the appropriate therapeutic option. In general, ^18^F-FDG PET/CT has higher specificity than CT, because ^18^F-FDG PET/CT diagnoses LN metastases on the basis of glucose metabolism rather than LN size [23]. Altini et al. reported that ^18^F-FDG PET/CT is a useful diagnostic tool for detecting primary lesions, LN metastasis, and distant metastases in gastric cancer [8]. ^18^F-FDG PET/CT is not always superior to CT; however, the sensitivity of ^18^F-FDG PET/CT is lower than that of CT (58.3% vs 70.8%), and the NPV is similar to that of CT (66.7% vs 65%] [8]. The lower sensitivity and similar NPV are due to false-negative or false-positive results, because ^18^F-FDG is not a tumor-specific tracer, and the glucose metabolism of tumors is affected by various factors, such as the histologic type and normal gastric walls [23, 24]. A tumor-specific tracer that can detect highly expressed antigens on gastric cancer cells could improve the NPV by decreasing the number of false negatives. Therefore, immuno-imaging targeting a tumor-specific antigen is expected to achieve higher NPVs and to result in more sensitive detection of LN metastases than is currently possible with ^18^F-FDG PET/CT in target antigen-positive tumors.

HER2-targeted PET imaging using ^89^Zr-trastuzumab has been investigated as an immuno-imaging method for gastric cancer. A clinical study showed a high degree of accumulation of ^89^Zr-trastuzumab in the foci of esophagogastric cancer patients [25], and a preclinical study using gastric cancer xenograft mice showed the highest tumor uptake of 34.4% ID/g at 96 h p.i. [26]. The number of patients well suited for PET imaging with ^89^Zr-trastuzumab is limited, however, because the positive ratio of HER2 is at most approximately 30% in gastric cancer, as demonstrated in the present study and previous studies [27]. On the other hand, the present study revealed higher positive ratios of CDH17 (85.7% [grade 2] and 51.9% [grade 3]) in gastric cancer. Therefore, compared with HER2-targeted imaging, CDH17-targeted imaging could be useful in a larger number of patients. CDH17 is expressed in not only gastric cancer but also colon cancer and pancreatic cancer: the expression ratios of colonic adenocarcinoma, pancreatic ductal adenocarcinoma, pancreatic adenocarcinoma, and cholangiocarcinoma were reported to be 97.3%, 39.3%, 24.1%, and 53.3%, respectively [9, 10, 28]. These findings suggest that imaging with ^111^In-D2101 could visualize these cancers in addition to gastric cancer. However, CDH17 is expressed in several normal organs such as the intestine. There is a need for further investigation to evaluate tumor-to-normal tissue ratios in non-gastric cancer.

There are several reports which show clinical relevance and biological function of CDH17 in gastrointestinal cancer. It is reported that CDH17 knockdown inhibits the growth of gastric cancer cells by downregulating Wnt/β-Catenin signaling [29]. In the animal study of intratumoral injection of CDH17 shRNA for gastric cancer xenograft mice, the growth of tumors injected with CDH17 shRNA was suppressed compared with control. Therefore, CDH17 is a promising therapeutic target for CDH17-positive gastric cancer. However, the therapy would require identifying the localization of cancer in vivo. CDH17-targeted imaging using ^111^In-D2101 is useful for the localization diagnosis. CDH17 is also reported as a prognostic factor for gastric cancer patients [29]. Median overall survival was 15.9 and 26.6 months in CDH17-positive and CDH17-negative gastric cancer patients, respectively. In addition, it was observed poor overall survival in patients with high expression of CDH17 as compared to patients with low expression of CDH17. Therefore, CDH17-targeted imaging using ^111^In-D2101 would be promising for prognostic prediction of gastric cancer patients.

The present study demonstrated that CDH17 is frequently expressed in both primary and metastatic gastric cancer, and that ^111^In-D2101 has high binding affinity in vitro, high accumulation in CDH17-positive tumors in vivo, and can be visualized by SPECT/CT imaging. The present study has several limitations, however, as follows: first, the uptake in CDH17-positive organs such as intestine may interfere distinguishing metastasis in lymph nodes around the stomach. Our study cannot completely predict the pharmacokinetics of our imaging probe radiolabeled D2101 in humans, because our antibody does not have cross-reactivity to murine CDH17. Therefore, there is a need for further clinical studies or preclinical studies with genetically engineered mice expressing human CDH17. Second, CDH17 expression analysis in gastric cancer specimens was performed with only a small sample size; therefore, further expression analyses using larger numbers of specimens are needed to confirm a role of CDH17-targeted imaging on N-staging in gastric cancer. Third, although ^111^In-D2101 showed high levels of accumulation in AGS tumors, a more sensitive imaging technique might be needed to detect very small LN metastases. Compared with SPECT, PET has higher sensitivity [30], and the development of D2101 labeled with a positron emitter having an appropriate half-life for antibody-based imaging, such as ^89^Zr, might be promising.

## Conclusion

Our anti-CDH17 Mab D2101 radiolabeled with ^111^In exhibited high levels of tumor uptake and low background levels in the biodistribution and SPECT/CT studies in a CDH17-positive gastric cancer mouse model. The immunostaining analysis demonstrated that the CDH17-positive ratio was higher than the HER2-positive ratio in gastric cancer specimens of both primary lesions and LN metastases. Therefore, CDH17 is a promising diagnostic marker, and radiolabeled D2101 has the potential to provide useful information for the N-staging of gastric cancer. These findings warrant further clinical studies to clarify the role of CDH17-targeted imaging in the diagnosis of gastric cancer.

## Abbreviations

Mab: Monoclonal antibody
N-staging: Staging of lymph-node metastasis
EUS: Endoscopic ultrasound
CT: Computed tomography
SPECT: Single-photon emission computed tomography
PET: Positron emission tomography
FDG: ^18^F-Fluoro-2-deoxy-d-glucose
NPV: Negative predictive value
CDH17: Cadherin-17
CK20: Cytokeratin 20
CHX-A′′-DTPA: [(*R*)-2-Amino-3-(4-isothiocyanatophenyl)propyl]-*trans*-(*S*,*S*)-cyclohexane-1,2-diamine-pentaacetic acid
HPLC: High-performance liquid chromatography
LN: Lymph node
